# Web-Based eHealth to Support Counseling in Routine Well-Child Care: Pilot Study of E-health4Uth Home Safety

**DOI:** 10.2196/resprot.1862

**Published:** 2013-02-11

**Authors:** Mirjam Elisabeth Johanna van Beelen, Ineke Vogel, Tinneke Monique Jozef Beirens, Gitte Caroline Kloek, Paul den Hertog, Monique Désirée van der Veen, Hein Raat

**Affiliations:** ^1^Erasmus MC–University Medical CenterDepartment of Public HealthRotterdamNetherlands; ^2^Consumer Safety InstituteAmsterdamNetherlands; ^3^Centre for Youth and Family RijnmondRotterdamNetherlands

**Keywords:** child health services, eHealth, counseling, health care evaluation mechanisms, health promotion, Internet

## Abstract

**Background:**

Providing safety education to parents of young children is important in the prevention of unintentional injuries in or around the home. We developed a Web-based, tailored safety advice module to support face-to-face counseling in the setting of preventive youth health care (E-health4Uth home safety) in order to improve the provision of safety information for parents of young children.

**Objective:**

This pilot study evaluated a Web-based, tailored safety advice module (E-health4Uth home safety) and evaluated the use of E-health4Uth home safety to support counseling in routine well-child care visits.

**Methods:**

From a preventive youth health care center, 312 parents with a child aged 10-31 months were assigned to the E-health4Uth home safety condition or to the care-as-usual condition (provision of a generic safety information leaflet). All parents completed a questionnaire either via the Internet or paper-and-pencil, and parents in the E-health4Uth condition received tailored home safety advice either online or by a print that was mailed to their home. This tailored home safety advice was used to discuss the safety of their home during the next scheduled well-child visit. Parents in the care-as-usual condition received a generic safety information leaflet during the well-child visit.

**Results:**

Mean age of the parents was 32.5 years (SD 5.4), 87.8% (274/312) of participants were mothers; mean age of the children was 16.9 months (SD 5.1). In the E-health4Uth condition, 38.4% (61/159) completed the online version of the questionnaire (allowing Web-based tailored safety advice), 61.6% (98/159) preferred to complete the questionnaire via paper (allowing only a hardcopy of the advice to be sent by regular mail). Parents in the E-health4Uth condition evaluated the Web-based, tailored safety advice (n=61) as easy to use (mean 4.5, SD 0.7), pleasant (mean 4.0, SD 0.9), reliable (mean 4.6, SD 0.6), understandable (mean 4.6, SD 0.5), relevant (mean 4.2, SD 0.9), and useful (mean 4.3, SD 0.8). After the well-child visit, no significant differences were found between the E-health4Uth condition and care-as-usual condition with regard to the satisfaction with the information received (n=61, *P*=.51). Health care professionals (n=43) rated the tailored safety advice as adequate (mean 4.0, SD 0.4) and useful (mean 3.9, SD 0.4).

**Conclusions:**

Less than half of the parents accepted the invitation to complete a Web-based questionnaire to receive online tailored safety advice prior to a face-to-face consultation. Despite wide access to the Internet, most parents preferred to complete questionnaires using paper-and-pencil. In the subgroup that completed E-health4Uth home safety online, evaluations of E-health4Uth home safety were positive. However, satisfaction scores with regard to tailored safety advice were not different from those with regard to generic safety information leaflets.

## Introduction

Unintentional injury is a major cause of death among young children in Europe and the United States [1,2]. It is also a major source of morbidity and loss of quality of life [3,4]. The most common causes of child mortality and morbidity by injury in and around the home are drowning, poisoning, burns, and falls [1]. Parents can reduce the risk of injuries by applying various safety behaviors. However, necessary safety behaviors are still not taken by a large number of parents, causing unnecessary risk of injury of young children [5-7].

Many countries have installed preventive youth health care, which refers to various activities to improve and protect the health, growth, and development of young people, and also to prevent illness and disability in early life. These activities include a system of maternal and child health care, which serves children from birth to 18 years of age [8]. In the Netherlands, all parents are invited to attend regularly scheduled well-child visits at the preventive youth health care center, free of charge. During these well-child visits growth and development of the children is monitored and relevant health information and vaccinations are provided. In the Netherlands, around 93% of parents attend one or more well-child visits with their child under 4 years of age. The attendance rates may vary from circa 50% to 93% between the specific child-age related scheduled visits [9]. Parents receive health information on several topics, including information about nutrition, growth, and child home safety [10]. Currently this health information is provided to parents by using generic information leaflets that parents receive at regular well-child visits.

With the current strain on health care, greater efficiency is required. Providing health information through the Internet, as an additional source of information, might be beneficial in various ways. For example, tailored safety information can be provided to parents prior to a preventive youth health care visit, and the information gathered by the eHealth module regarding specific safety behaviors can be provided to the physician/nurse to enhance the efficiency of face-to-face counseling, as is done with regard to other health topics, such as nutrition and physical activity [11-14]. However the use of eHealth modules has not yet been evaluated in providing home safety information in the setting of day-to-day preventive youth health care. The application of eHealth in preventive youth health care provides the opportunity of giving individual, tailored information.

eHealth is a "broad, emerging field in the intersection of medical informatics, public health, and business, referring to health services and information delivered or enhanced through the Internet and related technologies" [15]. It is the use of information and communications, especially the Internet, to improve or enable health and health care [16]. eHealth could be used for providing information for parents on several health topics, including information to promote home safety. Because tailored information combined with counseling, which can be provided by using eHealth, is based on the personal situation, parents could find the information more useful than general information materials [17]. Furthermore parents could be inclined to change their behavior, when the information they receive is perceived as personally relevant [18,19].

However, eHealth is currently not extensively applied in preventive youth health care. We developed a Web-based, tailored safety advice module to support face-to-face counseling at preventive youth health care centers (E-health4Uth home safety) to provide safety information for parents of young children [7,20]. By using this eHealth module, parents can prepare for the next well-child visit at the preventive youth health care center, with regard to issues concerning the safety of the child at home [20]. In addition, the health care professional can evaluate the results of the E-health4Uth home safety module prior to or during each visit in order to improve communication with the parents [20]. It is unknown whether such Web-based, tailored information can be fitted within current daily practice, existing organizational goals, and parent-health care professional interactions, which is known to be important for such an eHealth approach to be successful [21].

This pilot study evaluates a Web-based, tailored safety advice module (E-health4Uth home safety) and evaluates the use of E-health4Uth home safety to support counseling in routine well-child care.

## Methods

### Sample and Setting

Physicians and nurses of 4 preventive youth health care centers situated in the Rotterdam area in the Netherlands participated in this study. These preventive youth health care centers were chosen because of their ongoing collaboration with the Erasmus University Medical Center in Rotterdam. In 2006 and 2007, parents (N=958) were invited to participate in the study one month before their regular well-child visit at the preventive youth health care center at child’s age 11, 14, 18, or 24 months. Parents received written information about the study and provided written or online informed consent (checkbox). The Medical Ethics Committee of the Erasmus Medical Center gave a "declaration of no objection" for this study (MEC-2004-256).

### Study Design

**Figure 1:**
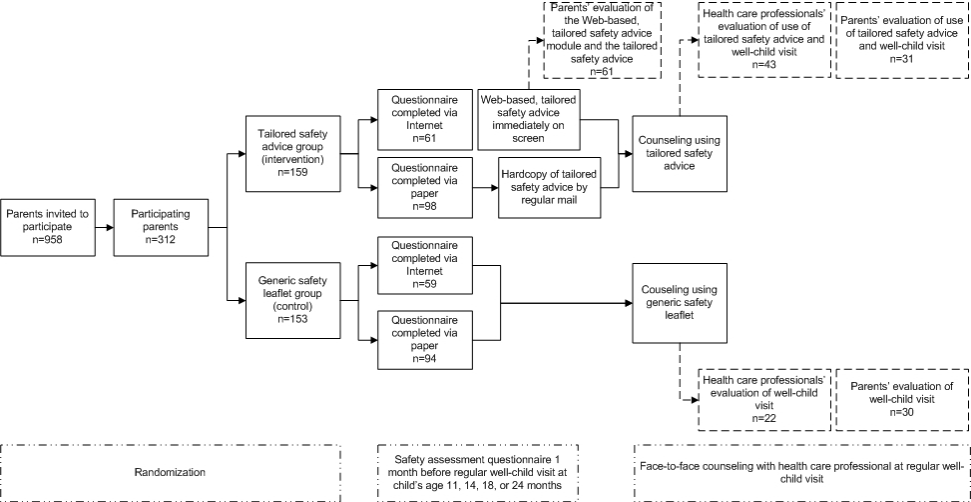
Study design, participant flow and evaluations.

Parents within each participating preventive youth health care center were randomly assigned to a Web-based, tailored safety advice and counseling group (ie, the E-health4Uth condition), or to a group receiving the generic safety information (ie, the care-as-usual condition, Figure 1).

#### E-health4Uth Home Safety Condition

A Web-based, tailored safety advice module (E-health4Uth home safety) was developed. Parents completed a self-report questionnaire (via the Internet or paper-and-pencil) to assess safety behaviors on the following safety topics: falls, poisoning, drowning, and burns [7]. When a situation was not applicable (eg, when parents did not have stairs in their house), they did not receive any more questions about that subject. After completing this safety assessment questionnaire parents received a tailored safety advice immediately on the screen when completed via Internet. The parents mailed the completed paper-and-pencil questionnaires back to the research center. The responses were entered into the database and a hardcopy of the resulting tailored safety advice was printed and sent to the parents via mail.

**Figure 2:**
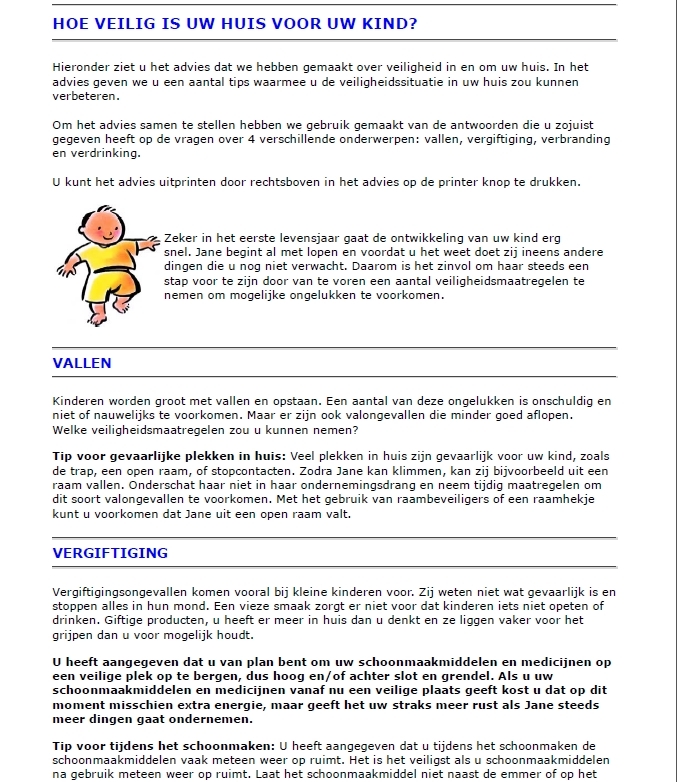
Sample page of tailored safety advice.

When parents failed to practice a particular safety behavior (“unsafe behavior”), they received a tailored message on how they can improve their safety behavior (Figure 2). When parents successfully showed a specific safety behavior (“safe behavior”) they received positive reinforcement and no safety advice on that item. The tailored safety messages were based on the guidelines of the Consumer Safety Institute [22]. Table 1 presents the contents and application of the tailored safety advices used in this study.

After completing the safety assessment questionnaire, parents were invited to visit the health care professional of their preventive health care center for their regular well-child visit. All the advice given to parents about safety were copied to the relevant health care professional, in order to enable the discussion of the advice with the parent during the visit. Parents and health care professionals could prepare for the well-child visit by formulating specific questions about the safety situation at home.

#### Care-as-Usual Condition

Parents of the care-as-usual condition completed the same self-administered questionnaire assessing parents’ child safety behaviors, either by using the Internet or paper-and-pencil. However they did not receive any tailored safety advice after completing the questionnaire. After completing the safety assessment questionnaire, parents visited the health care professional of their preventive health care center for a regular well-child visit. Parents received a generic safety information leaflet from their health care professional during their regular well-child visit (care-as-usual) [23,24]. This generic safety information leaflet was developed by the Consumer Safety Institute [22]. Each age group was provided with a different information leaflet, divided into 0-6 months, 6-12 months, 1-2 years, and 2-4 years of age. The safety information leaflets contained information on the prevention of injuries in and around the home, divided into general information about the development and environment of the child, and safety advice about the prevention of falls, poisoning, drowning, and burns.

**Table 1:** Contents and application of the tailored safety advice in the prevention of falls, poisoning, drowning, and burns.

		Applicable if:	Reinforcement with no tailored safety advice, when:	Tailored safety advice when:
**Prevention of falls**				
	Stair gate	The house has a staircase which the child can reach	A stair gate is present and is closed at all times	No stair gate is present A stair gate is present but is not closed at all times
	Balcony	The house has a balcony	The child is never left alone on the balcony	The child is left alone on the balcony
**Prevention of poisoning**				
	Cleaning products	Always		
	Medicines	Always	Stored above adult chest-height or in a locked cupboard	Stored below adult chest height or in an unlocked cupboard
**Prevention of drowning**				
	Bath tub	The child takes a bath	Child is never left alone in the bath tub	Child is left alone in the bath tub
	Swimming pool	The child swims in the swimming pool	Never left alone in the swimming pool	Child is left alone in the swimming pool
	Pond	There is a pond in the garden	Always the advice is to fill up the pond	A pond is present
**Prevention of burns**				
	Thermostat-controlled tap	Always	Thermostat-controlled tap present in the bathroom	Thermostat-controlled tap not present in the bathroom
	Hot drinks	Always	Child is never on parent’s lap when drinking hot liquids	Child on parent’s lap when drinking hot liquids
	Kitchen	Always	Child is never in the kitchen when the parent is cooking Parent cooks on the back griddle Handles of pans are turned to the back during cooking	Child is in the kitchen when the parent is cooking Parent does not cook on the back griddle Handles of pans are not turned to the back during cooking

### Measures

Socio-demographic data and parents’ safety behaviors were collected through a self-administered assessment questionnaire completed by the parents. Immediately after receiving the tailored safety advice, the parents (only those in the E-health4Uth condition who used the Internet to complete the questionnaire) were invited to complete a Web-based evaluation form about the tailored safety advice received and the use of the tailored safety advice module.

When parents attended the scheduled well-child visit, they were invited to complete an evaluation form about the well-child visit. Parents in the E-health4Uth condition were specifically asked about the use of tailored information during the face-to-face counseling. The youth health care professionals were also invited to complete evaluation forms regarding the well-child visit, and, if applicable, the use of the tailored information during the face-to-face counseling.

Evaluation items of the tailored safety advice, the Web-based tailored safety advice module, and the well-child visit, were measured on 5-point Likert scales ranging from 1 (most negative evaluation) to 5 (most positive evaluation), unless stated otherwise.

#### Evaluation of the Web-Based Tailored Safety Advice Module (Immediately After Receiving the Tailored Safety Advice)

Parents of the E-health4Uth condition who completed the Internet version of E-health4Uth home safety, were invited to complete a Web-based questionnaire after having read the tailored safety advice. The questions were: (1) reading of the safety advice (ie, having read the advice completely, partly or not at all), (2) the reliability, understandability, relevance, and usefulness of the tailored safety advice, (3) the ease of use of the module, and (4) the pleasantness of the information source.

**Table 2:** Characteristics of all parents and by E-health4Uth condition and care-as-usual condition (n=312).

			Total participants n (%)	E-health4Uth condition n (%)	Care-as-usual condition n (%)	*P* value
**Family characteristics**						
	Mean age of respondent in years (range, SD)		32.5 (20-48, SD 5.4)	32.3 (20-48, SD 5.7)	32.8 (20-44, SD 5.1)	.41^a^
	Mother is respondent		274/312 (87.8)	139/159 (87.4)	135/153 (88.2)	.83
	Non-Dutch mother		83/312 (26.6)	43/159 (27.0)	40/153 (26.1)	.83
	Non-Dutch father		82/312 (26.3)	45/159 (28.3)	37/153 (24.2)	.53
	Educational level of the respondent is low^b^		54/312 (17.3)	32/159 (20.1)	22/153 (14.4)	.14
	Single parent		30/312 (9.6)	15/159 (9.4)	15/153 (9.8)	.82
	One child in family		158/312 (50.6)	84/159 (52.8)	74/153 (48.4)	.71
**Child characteristics**						
	Mean age of child in months (range, SD)		16.9 (10-31, SD 5.1)	17.4 (10-30, SD 5.0)	16.5 (10-31, SD 5.3)	.14^a^
	Gender child, boys		154/312 (49.4)	80/159 (50.3)	74/153 (48.4)	.73
	Child can crawl		303/311 (97.4)	154/158 (97.5)	149/153 (97.4)	.96
	Child can pull up to standing		288/311 (92.6)	145/158 (91.8)	143/153 (93.5)	.57
	Child can walk independently		221/311 (71.1)	117/158 (74.1)	104/153 (68.0)	.24
	Child can climb		211/271 (77.9)	108/137 (78.8)	103/134 (76.9)	.70
**Safe and unsafe behaviors**						
	**Risk of falls**					.27
		Safe behavior	198/312 (63.5)	99/159 (62.3)	99/153 (64.7)	
		Unsafe behavior	90/312 (28.8)	44/159 (27.7)	46/153 (30.1)	
		Not applicable^c^	24/312 (7.7)	16/159 (10.1)	8/153 (5.2)	
	**Risk of poisoning**					.62
		Safe behavior	198/312 (63.5)	103/159 (64.8)	95/153 (62.1)	
		Unsafe behavior	114/312 (36.5)	56/159 (35.2)	58/153 (37.9)	
	**Risk of drowning**					.03
		Safe behavior	190/310 (61.3)	90/158 (57.0)	100/152 (65.8)	
		Unsafe behavior	107/310 (34.5)	57/158 (36.1)	50/152 (32.9)	
		Not applicable^d^	13/310 (4.2)	11/158 (7.0)	2/152 (1.3)	
	**Risk of burns**					.03
		Safe behavior	14/312 (4.5)	11/159 (6.9)	3/153 (2.0)	
		Unsafe behavior	198/312 (95.5)	148/159 (93.1)	150/153 (98.0)	

^a^Mann-Whitney *U-*test

^b^Low educational level: intermediate secondary education or less

^c^Not applicable on falls; when no staircase and balcony is present

^d^Not applicable on drowning; when parents do not bath their child, parents do not go swimming with their child, and no pond is present

#### Health Care Professionals’ and Parents’ Evaluation of the Use of the Tailored Safety Advice During the Well-Child Visit

After the well-child visit, health care professionals reported the duration of the visit for both conditions on their evaluation form.

Furthermore, health care professionals were invited, directly after each face-to-face consultation, to complete items regarding the following topics: (1) adequacy of the generated tailored safety advice, (2) usefulness of the tailored safety advice during the well-child visit, (3) the rating for the application of the tailored safety advice on a scale from 1 (most negative evaluation) to 10 (most positive evaluation), (4) whether the information of the tailored safety advice was in accordance with what the parent indicated (yes/no), (5) health care professionals’ satisfaction with the information given to the parent, and (6) health care professionals’ overall satisfaction with the well-child visit. Health care professionals rated both the use of the tailored safety advice and the well-child visit on a scale from 1 (most negative evaluation) to 10 (most positive evaluation).

In both the E-health4Uth condition and the care-as-usual condition, all parents that attended the scheduled well-child visit, at the end of the visit, were invited to complete items regarding the satisfaction with the safety information they received (tailored or generic), the overall satisfaction with the well-child visit, and the rating for the well-child visit on a scale from 1 (most negative evaluation) to 10 (most positive evaluation). Parents in the E-health4Uth condition completed an additional item on whether discussing the tailored safety advice was a valuable supplement.

Furthermore both parents in the E-health4Uth condition and the care-as-usual condition had to report their intention to change safety in or around the home after the well-child visit (ie, prevention of falls, poisoning, drowning, and burns: yes/no).

### Statistical Analysis

Statistical analyses were performed using SPSS 17.0 (SPSS Inc., Chicago, IL, USA).

Frequency tables were used to explore the socio-demographic characteristics of the total study population and of both conditions (E-health4Uth and care-as-usual). The frequency of safe and unsafe behavior on each safety topic was determined. The differences were examined with chi-square tests. Items about the well-child visit and the intention to change safety behavior after the well-child visit were compared between the current method of providing safety information (care-as-usual condition) and the tailored safety advice (E-health4Uth condition). Differences were determined with student’s *t* tests. Mann–Whitney *U* tests were used to assess data that were not normally distributed.

## Results

### Family and Child Characteristics

A total of 312 parents (312/958, 32.6%) provided informed consent and participated in the study—159 parents were assigned to the E-health4Uth condition and 153 to the care-as-usual condition. The mean age of the parents was 32.5 (range 20-48, SD 5.4) years, 87.8% (274/312) of parents were mothers and 17.3% (54/312) of parents had a low educational level (intermediate secondary education or lower). In this study, 90.4% (282/312) of responding families included both parents and 50.6% (158/312) of families had one child. The age of the children ranged from 10-31 (mean 16.9, SD 5.1) months, 49.4% (154/312) of children were boys. Almost all children could crawl (303/312, 97.4%), and 92.6% (288/312) could pull up to standing (Table 2). Unsafe behaviors related to risk of falls were performed by 28.8% (90/312) of the parents, while 36.5% (114/312) of parents performed unsafe behaviors with regard to risk of poisoning, 34.5% (107/312) with regard to drowning, and 95.5% (298/312) of parents performed unsafe behaviors with regard to risk of burns. More parents in the E-health4Uth condition performed unsafe behaviors with regard to the risk of drowning compared to the care-as-usual condition (36.1% vs 32.9%, *P*=.03). More parents in the care-as-usual condition performed unsafe behaviors with regard to the risk of burns compared to parents in the E-health4Uth condition (98.0% vs 93.1%, *P*=.03).

In the E-health4Uth condition, 38.4% (61/159) completed the online version of the questionnaire (allowing Web-based tailored safety advice), while 61.6% (98/159) preferred to complete the questionnaire via paper (allowing only a hardcopy of the advice to be sent by regular mail).

#### Evaluation of the Web-Based Tailored Safety Advice Module (Immediately After Receiving the Tailored Safety Advice)

All of the parents in the E-health4Uth condition who completed the questionnaire via the Internet completed the Web-based evaluation of the safety advice and the safety advice module, directly after receiving the advice (n=61). Of these, 82.0% (50/61) of parents reported having read their safety advice completely, 13.1% (8/61) of parents read the advice only partly, and 4.9% (3/61) of parents (5%) did not read the advice at all (Table 3). Parents considered the tailored safety advice to be reliable (mean 4.6, SD 0.6), understandable (mean 4.6, SD 0.5), relevant (mean 4.2, SD 0.9), and useful (mean 4.3, SD 0.8). Furthermore, these parents evaluated the Web-based, tailored safety advice module as easy to use (mean 4.5, SD 0.7) and found it a pleasant information source (mean 4.0, SD 0.9).

#### Health Care Professionals’ and Parents’ Evaluation of the Use of the Tailored Safety Advice During the Well-Child Visit

We received 65 evaluation forms completed by health care professionals with regard to the well-child visits (43 in the E-health4Uth condition, 22 in the care-as-usual condition) and we received 61 evaluation forms from parents who attended the scheduled preventive youth health care visit (31 in the E-health4Uth condition, 30 in the care-as-usual condition).

The mean duration of the well-child visit, as reported by the health care professionals, was 27.2 minutes (SD 11.1) in the E-health4Uth home safety E-health4Uth condition versus 23.7 (SD 8.0) minutes in the care-as-usual condition (*P*=.32).

Health care professionals who completed and submitted the evaluation forms regarding the well-child visits found discussing the tailored safety advice with the parents to be adequate (mean 4.0, SD 0.4) and useful (mean 3.9, SD 0.4, Table 4). They rated the application of the advice positively (mean 7.3, SD 1.0). Eighty-one percent (29/36) of youth health care professionals reported that the information found in the tailored safety advice was in accordance with what the parent indicated. Health care professionals were satisfied with the information they gave to the parents of both conditions (mean 4.1, SD 0.6 for the E-health4Uth condition and mean 4.3, SD 0.5 for the care-as-usual condition, *P*=.31), and there was also no difference in overall satisfaction of the well-child visit between the E-health4Uth condition and care-as-usual condition (*P*=.16). Health care professionals rated the well-child visit with parents in the care-as-usual condition slightly higher than that with parents in the E-health4Uth condition (ie, mean 7.8, SD 0.8 vs mean 7.5, SD 0.9 respectively, *P*=.23).

Among parents that attended the scheduled well-child visit and who completed the evaluation forms, parents of both the E-health4Uth condition and care-as-usual condition were satisfied with the information received during the well-child visit (mean 3.7, SD 0.8 and mean 3.4, SD 1.3, respectively). Discussing the tailored safety advice with the youth health care professional was a valuable supplement to the well-child visit (mean 3.4, SD 1.3). No significant difference was found in satisfaction between the E-health4Uth condition and care-as-usual condition (*P*=.51). Parents in both the E-health4Uth condition and care-as-usual condition gave the well-child visit a mean rating of 8.0.

More parents in the E-health4Uth condition showed intentions to change safety in or around the home with regard to the prevention of falls (43.3% vs 18.5%, *P*=.04), the prevention of poisoning (53.6% vs 29.6%, *P*=.07), the prevention of drowning (35.7% vs 14.8%; *P*=.08), and the prevention of burns (57.1% vs 22.2%, *P*=.008) compared to parents in the care-as-usual condition.

**Table 3:** Parents’ evaluation of the tailored safety advice and the Web-based, tailored safety advice module (n=61).

**Reading of the Web-based, tailored safety advice**		**n (%)**
	Have read their advice completely	50/61 (82.0)
	Have read their advice partly	8/61 (13.1)
	Have not read their advice	3/61 (4.9)
**Tailored safety advice**		**Mean (SD)**
	Was the safety advice reliable?^a^	4.6 (0.6)
	Was the safety advice understandable?^a^	4.6 (0.5)
	Was the safety advice relevant?^a^	4.2 (0.9)
	Was the safety advice useful?^a^	4.3 (0.8)
**Web-based, tailored safety advice module**		
	Was the module easy to use?^a^	4.5 (0.7)
	Was the module a pleasant information source?^a^	4.0 (0.9)

^a^ Scores on a 5-point Likert scale ranging from 1 (most negative evaluation) to 5 (most positive evaluation)

**Table 4:** Health care professionals’ and parents’ evaluation of the well-child visit and the use of the tailored safety advice during the well-child visit (if applicable).

		E-health4Uth condition	Care-as-usual condition	*P* value
**Health care professionals:**		**Mean (SD)** **n=43**	**Mean (SD)** **n=22**	
	Was discussing the safety at home adequate?^a^	4.0 (0.4)	NA^c^	
	Was the tailored safety advice useful to discuss during the well-child visit?^a^	3.9 (0.4)	NA^c^	
	Rating for the application of the tailored safety advice^b^	7.3 (1.0)	NA^c^	
	Was the information of the tailored safety advice in accordance with what the parent indicated? n (%)	29/36 (80.6%)	NA^c^	
	Satisfaction with information given^a^	4.1 (0.6)	4.3 (0.5)	.31^d^
	Overall satisfaction with the well-child visit^a^	4.2 (0.4)	4.3 (0.4)	.16^e^
	Rating for the well-child visit^b^	7.5 (0.9)	7.8 (0.8)	.23^d^
**Parents:**		**Mean (SD)** **n=31**	**Mean (SD)** **n=30**	
	Satisfaction with information discussed^a^	3.7 (0.8)	3.4 (1.3)	.51^d^
	Was discussing the tailored safety advice valuable supplement?^a^	3.4 (1.3)	NA^c^	
	Overall satisfaction with the well-child visit^a^	4.4 (0.5)	4.4 (0.5)	.92^d^
	Rating for the well-child visit^b^	8.0 (1.2)	8.0 (0.9)	.92^d^
**Intention to change safety behavior in or around the home after the well-child visit, in the prevention of:**		**n (%)**	**n (%)**	
	Falls	13/30 (43.3)	5/27 (18.5)	.04^f^
	Poisoning	15/28 (53.6)	8/27 (29.6)	.07^f^
	Drowning	10/28 (35.7)	4/27 (14.8)	.08^f^
	Burns	16/28 (57.1)	6/27 (22.2)	.008^f^

^a^Scores on a 5-point Likert scale ranging from 1 (most negative evaluation) to 5 (most positive evaluation)

^b^Scores from 1 (most negative evaluation) to 10 (most positive evaluation)

^c^Not Applicable

^d^Chi square

^e^Student’s *t-*test

^f^Mann-Whitney *U-*test

## Discussion

### Principal Results

In the present pilot study, we evaluated a Web-based tailored safety advice and the application of an eHealth module compared to the use of generic safety information leaflets in well-child visits. This pilot study showed that although the tailored safety advice and the E-health4Uth module turned out to be positively evaluated, the majority of parents declined to complete the online questionnaires that enabled online tailored safety advice, and preferred to use paper-and-pencil to complete the questionnaires. This diminishes the convenience of the use of Internet to deliver online tailored safety information. In the small subgroup of parents that attended the scheduled well-child visits and those that completed the evaluation form after the visit, the ratings regarding satisfaction in the E-health4Uth condition were equal to those in the care-as-usual condition, stating that parents have no preference with regard to the method of providing safety information during the well-child visit. However, among these parents, more parents in the E-health4Uth condition reported a favorable intention to change the safety situation in and around the home compared to parents in the care-as-usual condition.

### Limitations and Considerations

In this study, the participation rate (312/958, 32.6%) was relatively low. One reason for the low participation rate could be the lack of sending reminders. There is no data available on the characteristics of parents who did not wish to participate in this study. Baseline characteristics show that in the study population, over 90% of children were living in a two-parent home. In the general population of the Netherlands, the percentage of two-parents homes is comparable with the numbers we found in our study [25]. The relatively low participation rate might limit the generalizability of the results.

Slightly more parents in the E-health4Uth condition carried out unsafe behaviors with regard to the risk of drowning compared to the care-as-usual condition and slightly more parents of the care-as-usual condition behaved unsafe with regard to the risk of burns compared to parents of the E-health4Uth condition. Given the random allocation to both conditions, this was a chance finding.

All parents who provided informed consent completed the safety behaviors questionnaire, either by using Internet or by paper-and-pencil; and all parents in the E-health4Uth condition who completed the online questionnaire, provided answers to the evaluation form regarding the online tailored safety advice. However, relatively few evaluation forms from both parents (n=61) and professionals (n=65) were collected after the scheduled preventive youth health care visit. At the time of the study, there was no digital database regarding the preventive youth health care visits; so we were unaware whether the scheduled visits were realized or not. Furthermore, the empty form (that should have been completed) might have been missing in the dossier of the child (due to logistical problems), or the parent/professional did not want to complete the form. There was no significant difference in parent and child characteristics between parents who did and who did not complete the evaluation (*P*>.05, data not shown).

For the present study, since most well-child visits did not involve a vaccination (which is in the Netherlands associated with a high attendance), we might assume that circa 50% of the invited parents attended the scheduled visit. If this was the case, only circa 4/10 evaluation forms after the visits were collected. In future studies, we recommend the use of digital patient files to record attendance to the scheduled visits and the topics that were discussed during these visits. Brief evaluation questions may be integrated in such digital patient files with informed consent from the study participants. In the present study the results with regard to the evaluations after the preventive youth health care visits should be interpreted with utmost care, since non-response bias may have occurred, and given the relatively low numbers of completed forms. Furthermore, the evaluation of the well-child visit could depend on more items than just the ones we measured in this study.

Over 60% of parents preferred completing the safety behavior questionnaire by paper-and-pencil. In the E-health4Uth condition, this meant that less than 40% of participants could benefit from the online tailored safety advice. In this study, a hard copy of the tailored advice was generated after data-entry of the paper-and-pencil questionnaire results and mailed to both parent and health professional. This is however time consuming, costly, and diminishes the convenient nature of using the Internet to deliver online tailored safety information. According to Statistics Netherlands, the number of Internet connections in the Netherlands was 80% during the time of the study, rising to 94% in 2011 [25]. Lack of Internet connections does not fully explain why parents preferred to complete the health behavior questionnaire by using paper. Apparently, the majority of parents did not highly appreciate the possibility of online tailored advice. On the other hand, the parents that did complete the online version of E-health4Uth home safety read the advice and provided favorable ratings. We recommend, however, developing strategies to improve the uptake of eHealth applications and the perceived benefits of online tailored information in the practice of preventive youth health care by involving both parents and professionals.

One element may be to increase the perceived benefits from online tailored advice as opposed to current generic advice (most often provided as leaflets during well-child visits). This study showed, unexpectedly, a lack of difference between levels of satisfaction regarding tailored safety information provided between the E-health4Uth module and the generic safety information leaflet. We saw that parents were highly satisfied with both the current generic version as well as the tailored safety information, which implies that parents might not have a preference for either method. Safety information is only one topic in preventive youth health care. When the E-health4Uth module covers more relevant topics in the future, more advantage may be gained by providing tailored advice. We recommend involving both panels of parents and health professionals in such developments, in order to achieve maximum profit for the target audience of such eHealth tools. The current pilot study shows that a high uptake, let alone higher appreciation of tailored advice compared to high-quality generic advice cannot be taken for granted.

Although the difference was not statistically significant, the well-child visit lasted slightly longer in the E-health4Uth condition compared to the care-as-usual condition. The youth health care professionals reported a significantly longer duration of the visit in the E-health4Uth condition when a Web-based, online tailored information was generated and provided to both parent and professional (n=21, mean 31.4 minutes, SD 11.8) compared to when parents completed the questionnaire online and a hard copy of the advice was generated and provided to both parent and professional (n=22, mean 23.1 minutes, SD 8.8, *P*=.01, data not shown). For practical reasons we suppose the online generated tailored advice was more likely to be available at the moment of the health visit to both professional and parent, compared to the hard copy that had to be generated and mailed. Presence of the tailored advice might trigger more and longer discussions between parent and professional. Although this may be beneficial from the viewpoint of behavior goals to be attained, the duration of visits after provision of online tailored advice requires attention in future projects for logistical and financial reasons.

The current study, although in a relatively small, and potentially biased subgroup, illustrated that the tailored advice may induce more intention to change behaviors in a favorable direction. This supports favorable results from early initiatives [20,26]. To determine whether the Web-based tailored safety advice is more effective to promote parents’ child safety behaviors compared to generic advice using information leaflets, it is recommended that an effect-evaluation of E-health4Uth home safety is performed [27,28].

### Conclusions

There are many potential benefits of gathering health and health behavior data [29-31] and providing tailored information and support through eHealth in preventive youth health care. Online information sources and algorithms to generate tailored information can be easily updated, and wide-scale distribution can be arranged at relatively low cost. However, given the fact that the majority of parents did not accept the invitation to complete E-health4Uth home safety online and preferred paper-and-pencil instead, we recommend developing strategies to improve the uptake of eHealth applications and the perceived benefits of online tailored information in the practice of preventive youth health care by involving both parents and professionals. An example could be that parents are unaware of the benefits of tailored health information, due to lack of knowledge about this way of providing information. This lack of knowledge could lead to the fact that parents rather choose the regular approach in receiving health information, ie, with generic leaflets. Health care professionals could explain the goals and benefits to parents, so parents can better choose between these two forms of information provision. To examine why parents may not prefer an eHealth intervention, an overview of the advantages and disadvantages of the eHealth intervention by users could be collected, for instance how to deal with privacy issues.

Tailored information has the potential to be more effective in realizing favorable health behaviors compared to generic information, but not all potentially effective elements were already included in the prototype in this study. The current Web-based, tailored safety advice module and the use of the safety advice during the well-child visit could be extended using personal cognitive factors, social factors, or parents’ barriers to show safety behavior [32].

Changing behavior is difficult, requiring time, effort, and motivation. Health care professionals could benefit from techniques to help them motivate parents to change their behavior. Previous research has shown positive effects of motivational interviewing on health behavior [33-35]. Motivational interviewing provides techniques that can be applied by health care professionals to promote safe behavior. Motivational interviewing could be applied to the discussion of the tailored safety advice by the health care professional with the parent [36].

We propose future effect-evaluations of tailored safety advice, by exploring whether tailored safety advice is more effective on parents’ child safety behaviors compared to generic safety leaflets. When proven to be effective, eHealth combined with personal counseling could also be used in health promotion on multiple other areas relevant for prevention such as nutrition, physical activity, or sleep. It may be useful for parents to prepare themselves for the well-child visit and to formulate specific questions on these topics. Furthermore eHealth could help parents and youth health care professionals to focus on issues that need further attention.
